# Decrease Retinal Thickness in Patients with Chronic Migraine Evaluated by Optical Coherence Tomography

**DOI:** 10.3390/diagnostics13010005

**Published:** 2022-12-20

**Authors:** Isidoro Raga-Martínez, Francisco J. Povedano-Montero, Jesús Hernández-Gallego, Francisco López-Muñoz

**Affiliations:** 1 Faculty of Health Sciences, University Camilo José Cela, 28692 Madrid, Spain; 2 Centro Óptico Raga, 23700 Linares, Spain; 3 Hospital Doce de Octubre Research Institute (i+12), 28041 Madrid, Spain; 4 Faculty of Optics and Optometry, Complutense University, 28040 Madrid, Spain; 5 Centro Óptico Montero, 28032 Madrid, Spain; 6 Neurology Service, Hospital Universitario Doce de Octubre, 28041 Madrid, Spain; 7 Department of Medicine, Faculty of Medicine, Complutense University, 28040 Madrid, Spain

**Keywords:** retina, chronic migraine, optical coherence tomography, neurological disorder

## Abstract

The purpose of this study is to determine the possible alterations that may occur in the thickness of the retinal nerve fibre layer (RNFL), ganglion cell layer (GCL), and macular thickness in patients with chronic migraines compared with healthy controls. Hence, we examined some of the possibilities that are offered by optical coherence tomography (OCT) in order to study different neurological diseases and to study its application, in this case, how it may be applied to patients with chronic migraines. This was an observational cross-sectional study in adults aged 18–65 years. The study group consisted of 90 patients (90 eyes) with chronic migraines who met the inclusion criteria, and 90 healthy controls (90 eyes) matched for age and sex. Retinal thickness was measured by spectral domain OCT (SD-OCT). The thickness of the superior quadrant of the peripapillary RNFL, as well as the mean thickness in the macula, RNFL macular, and GCL was significantly thinner in chronic migraine patients than in healthy controls (*p* ≤ 0.05). Chronic migraines are associated with a decrease in retinal thickness which is detectable by an OCT diagnostic technique. The quantification of the axonal damage could be used as a biomarker to help in the diagnosis and monitoring of this pathology. Further studies will be needed to confirm these findings.

## 1. Introduction

A migraine is a frequent, disabling, and almost always episodic primary headache, the main symptom of which is a pulsating, unilateral headache, associated with nausea or vomiting, and a sensitivity to light and sound. Many epidemiological studies have documented its high prevalence and enormous socio-economic and personal impact [1,2,3]. According to the Global Burden of Disease Survey 2010 (GBD2010) it is the third most prevalent disorder in the world. In the GBD2015, it was classified as the third cause of disability in both men and women under 50 years of age worldwide [4]. If it is preceded by neurological symptoms, it is classified as migraine with aura, 99% of which are of a visual nature, and if the migraine lasts for more than fifteen days a month, it is considered a chronic migraine (CM).

Migraines are associated with a multitude of comorbid diseases and disorders, such as metabolic syndrome. More specifically, there is relationship between CM and obesity [5], blood pressure abnormalities [6], and systolic hypertension [7,8]. Although it is not clear how CM and metabolic syndrome interact, there is a consensus that preventive treatments and good habits such as weight loss, good sleep hygiene, and physical activity, can help prevent the development of metabolic syndrome, and therefore, benefit patients with CM.

The mechanisms that cause a person to be susceptible to migraine episodes are not fully understood. Previous studies agree that there is an autosomal dominant pattern of inheritance in up to 98% of cases [9]. Although it occurs more frequently in women than in men, there are numerous genes involved, and hence, there are a variety of symptomatologies presented by patients.

In the pathophysiology of a migraine, activation and sensitisation occurs episodically in the trigeminal vascular system [10,11], which is also found in extracranial structures, such as the retina and choroid. Its activation causes inflammation, in addition to causing vasodilation and subsequent vasoconstriction in both structures. It is known that this vascular narrowing and the lack of blood supply is temporary, both at the cerebral and ocular level, but the chronic nature of migraine may be the cause of permanent structural and functional alterations [12,13,14]. All these retinal changes can be measured by optical coherence tomography (OCT).

OCT is a non-invasive imaging technique based on the principle of low-coherence light interferometry. This technique uses infrared light reflection to obtain micrometre-scale tomographic images and it is used to analyse biological tissue structures [15]. The light reflected from the tissue produces cross-sectional images with an axial resolution of less than 10 microns [16].

In the case of the retina, OCT provides relevant information for the diagnosis and study of the pathogenesis of a wide variety of conditions associated with retinal tissue degeneration [16], and thus it is able to obtain accurate and reproducible measurements of the retinal nerve fibre layer (RNFL), macular volume, and ganglion cell layer (GCL) [17,18].

OCT is increasingly used to study the anterior segment [19,20] and the papilla in glaucoma [21,22,23], although the study of the retina, and especially the macula [24,25,26], remains to be its main application. The obtained tomographic images allow the diagnosis of pathologies that are difficult to identify ophthalmologically. Furthermore, the ability to scan the same area of the retina on different occasions makes it possible to monitor the retina [27].

As it is an extension of the central nervous system [28,29], the retina has attracted the interest of neuroscientists as it acts as a window into pathological processes in the brain. OCT has been used, over the last few years, in multiple studies that have demonstrated scientific evidence of RNFL thinning and GCL alterations in various neurological diseases where some kind of nervous system disorder occurs. The results of these studies indicate that there is a correlation between RNFL thickness, brain atrophy, and clinically, visual dysfunction, thus allowing the eye to be a model for studying neurodegenerative diseases [30,31,32,33] such as Parkinson’s disease, Alzheimer’s disease, and multiple sclerosis. Evidence has also been found in other diseases such as epilepsy [34] and substance abuse disorders such as alcohol dependence [35]. In 2008, Martínez et al. published the first study on RNFL thickness reduction in migraine patients [36].

In this study, we compared the thickness of different retinal layers between chronic migraine patients and healthy controls using spectral domain OCT (SD-OCT). The unique aspect of our work is that we have focused on the study of CM, unlike most of the research carried out to date which has mainly studied non-specific migraines. In addition, the measurement of GCL and RNFL was performed in two different segmentations: GCL in GCL++ and GCL+, and RNFL in macular and peripapillary. These segmentations are described in the methods section. We also aimed to find a correlation between morphological alterations in the retinas of patients who experienced years of CM, as well as a relationship between possible alterations in the retina and the laterality of migraine pain. Considering the migraine–eye relationship, as discussed above, it was hypothesised that by comparing them to healthy patients, chronic migraine patients may show alterations in RNFL thickness, macular thickness, and GCL.

## 2. Materials and Methods

### 2.1. Study Sample

An analytical and observational cross-sectional study was conducted in a group of subjects diagnosed with CM and another group of healthy controls adjusted for age and sex. These subjects were subjected to a non-invasive exploration, with SD-OCT, to determine the possible existence of alterations in the RNFL, the GCL, and the macula. In both groups, participants were Caucasian and over the age of 18. The patients were recruited at the Neurology Clinic of the Hospital 12 de Octubre in Madrid (Spain) and at the Optometry Clinic of the Centro Óptico Raga in Linares (Jaén, Spain), thus fulfilling the requirement of being diagnosed with CM by a medical specialist, in accordance with the criteria of the International Headache Society [4]. The antecedents and detailed medical history of the patients were recorded. Likewise, information was obtained that pertained to the frequency of migraine attacks and their duration, the lateralisation of pain, age at migraine onset, years of migraine evolution, presence or absence of aura, and the medication that patients were taking for both the prevention and treatment of CM. The MIDAS (Migraine Disability Assessment Scale) questionnaire was also used to assess how CM affected quality of life in the preceding 3 months [37,38].

Each participant was informed about the study, and they signed an informed consent form before participation in the study. A standard ophthalmological examination was performed, including determination of refractive error and best-corrected visual acuity, as well as an ocular pressure assessment with air-puff tonometry.

The patients were excluded if they were suffering from any neurological disease, cognitive impairment, or major chronic disease (such as diabetes mellitus, uncontrolled hypertension or low blood pressure, dyslipidemia, coagulation disorders, cardiopathy), or if they were taking any medication that could affect retinal thickness, such as drugs with a vasoconstrictive effect (e.g., triptans). Other criteria for ophthalmological exclusion included: suffering from any ophthalmological disease, ocular hypertension (>20 mg Hg), or history of glaucoma, and ocular surgery (with the exception of cataract surgery). The exclusion limit based on the refraction refractive error was 5 diopters of spherical equivalent refraction and/or 3 diopters of astigmatism.

Approval of the study was requested from the Research Ethics Committee of the Camilo José Cela University (UCJC), which provides certification to confirm that the study is subject to the institutional ethical provisions of the UCJC and the Declaration of Helsinki.

### 2.2. Procedures

The OCT device used was the Topcon 3D OCT-1 Maestro (Topcon, Itabashi-ku, Tokyo, Japan), firmware version 1.29, which is a SD-OCT that combines a high-resolution colour fundus camera with Fourier domain OCT technology, and it provides a high capture rate of 50,000 A-Scans/sec (images, axially, acquired per second). The resolution of the device is 20 μm in lateral resolution and 6 μm in-depth resolution. The accuracy of the OCT is 0.76 μm [39,40].

Macular thickness, peripapillary RNFL, RNFL macular, and GCL were evaluated with this SD-OCT device. The scans, for all participants, were performed under the same dim lighting conditions and at the same time of the day (measured between 10 a.m. and 12 a.m.), in order to minimise the effects of diurnal variation. The scans were conducted by the same experienced technician.

An internal fixation target was used in all the scans, with a fully automated alignment, focusing, and capture system, plus real-time eye tracking. It also self-centred on fovea and papilla in order to adjust to eye movements.

**Figure 1:**
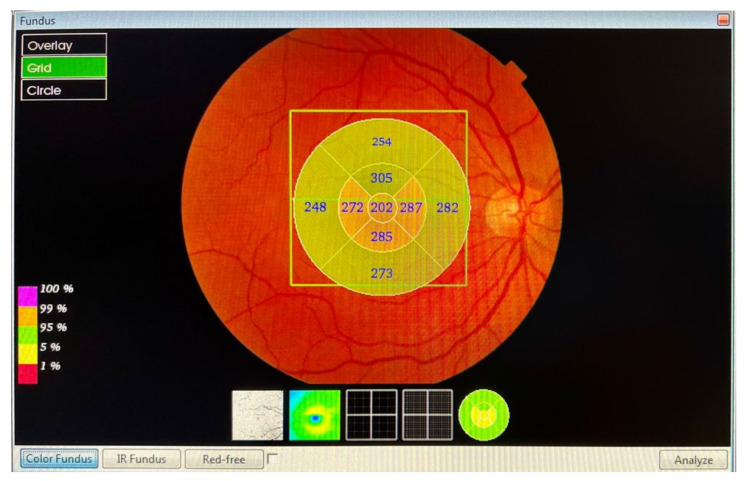
The *3D Macula Report* protocol.

The macular and RNFL thickness in the macula, measured in microns, was automatically determined and analysed by the OCT software *Fast Map v.8.40* using the *3D Macula Report* protocol. The evaluation of the macular thickness, using the software, included a 6.0 × 6.0 mm (horizontal/vertical) scan, with a resolution of 512 × 128 pixels, which was centred on the fovea, and configured in nine quadrants. The *3D Macula Report* protocol (Figure 1) after macular map analysis displayed the numerical averages of the measurements for each of the nine quadrants, as defined by the ETDRS circular grid. The diameters of the concentric circles were 1, 3, and 6 mm for the macular scan. This software provided a thickness profile of the:

- Macula: from the inner limiting membrane (ILM) to the retinal pigment epithelium (RPE), the profile included, in nine ETDRS quadrants, the superior peripheral, superior, central, inferior, inferior peripheral, temporal peripheral, temporal, and nasal peripheral. The profile centred on the fovea.
- RNFL macular: from the ILM to RNFL, the profile included, in nine ETDRS quadrants, the superior peripheral, superior, central, inferior, inferior peripheral, temporal peripheral, temporal, and nasal peripheral. The profile focused on the fovea.

**Figure 2:**
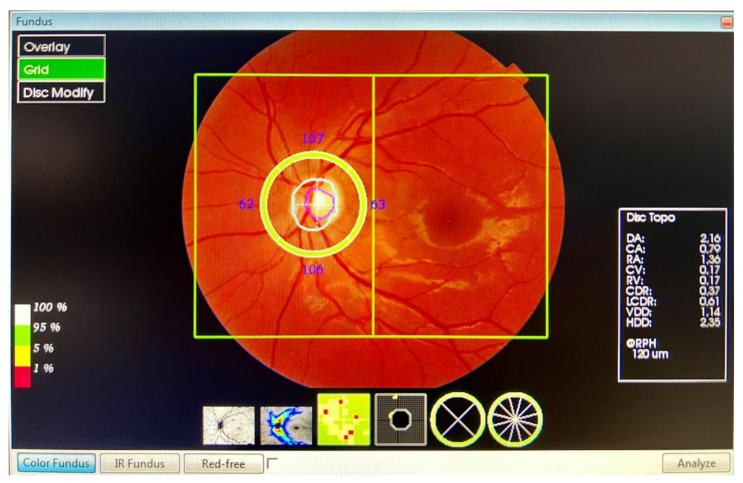
The *3D Wide Report* protocol.

The peripapillary thickness parameters of the RNFL, GCL++ (RNFL + GCL + inner plexiform layer (IPL)), and GCL+ (GCL + IPL) were automatically calculated using the *3D Wide Report* protocol (Figure 2). A 12 × 9 mm (horizontal/vertical) scanner with a 512 × 128 pixel resolution, together with a segmentation, using automated algorithms of six retinal layers (from the ILM to the RPE), provides the measurement and topographical maps of the optic nerve and macula in a single scan. This software provided a thickness profile of the:

- Peripapillary RNFL: from ILM to the GCL, in four quadrants: superior, inferior, nasal, and temporal. The profile centred on the optic nerve.
- GCL++: from ILM to the IPL, in six ETDRS quadrants: superior, superior temporal, superior nasal, inferior, inferior temporal, and inferior nasal. The profile centred on the fovea.
- GCL+: from GCL to the IPL, in six ETDRS quadrants: superior, superior temporal, superior nasal, inferior, inferior temporal, and inferior nasal. The profile centred on the fovea.

For the study of the GCL, we divided the analysis into the ganglion cell complex (GCC), which includes the profile that ranges from the ILM to the IPL, which we called GCL++, and the GCL + IPL (GCIP), which we called GCL+.

Figure 1 and Figure 2 show the scanned images that were obtained from two of our subjects.

The 3D OCT-1 Maestro uses a signal-to-noise estimation (SNR in dB) to indicate the quality of the measurements made. After all the exposures were carried out, the non-centred scans, or those with SNR < 20 dB, were excluded from the study. The analysis report also provided a description of the percentile (≤95° to >5°, normal, ≤5° to 1°, borderline, and <1°, abnormal) in which the RNFL thickness was located, in accordance with the manufacturer’s database of age-corrected normal values.

### 2.3. Sample Size Calculation

The statistical programme G*Power 3.1 was used to calculate the sample needed to detect possible differences between independent groups (study patients and control subjects) in relation to the thinning of the different retinal layers. Assuming one case per control, and in accordance with previous studies [41], with the expectation of a mean difference or effect size of 0.38 between cases and controls, 87 CM patients and 87 control subjects would be needed to obtain a confidence level of 95%, an alpha risk (∝) = 0.05, a statistical power of 80%, and a beta risk (β) = 0.20.

### 2.4. Statistical Analysis

Statistical analyses were performed using the IBM SPSS Statistics version 25 for Mac software (SPSS Inc. Chicago, IL). First, the mean value and standard deviation were calculated for the whole set of variables measured. To assess the behaviour of the data obtained, the Kolmogorov–Smirnov test was applied for a significance level of 5%, which was considered to be normal when the *p*-value associated with the statistic was greater than 0.05. Since the data followed a normal distribution, parametric tests were applied.

To assess whether there were significant differences between the two samples, the Student’s *t*-test for independent samples was applied. A *p*-value of 0.05 was considered to be statistically significant. As there were multiple comparisons, the Bonferroni correction was applied. This involved dividing the *p*-value by the number of variables studied in each case: for the macula, 10 variables (*p* ≤ 0.005); for the optic nerve, 5 variables (*p* ≤ 0.01); and for the GCL+ and GCL++, 7 variables (*p* ≤ 0.007). Only one eye per patient was chosen randomly for the study.

To determine a possible relationship between the years of development of the disease and the thicknesses obtained in the different layers, the Pearson correlation coefficient test was performed on those layers where the greatest differences in thickness were found between the patients and controls, namely, the macula and GCL++.

Finally, to find a relationship between CM laterality and retinal thickness difference in each eye, a one-factor ANOVA test with Tukey’s post-hoc correction for multiple comparisons (*p* ≤ 0.05) was used.

The statistical programme G*Power 3.1 was also used for all variables that were significant at *p* ≤ 0.05, in order to calculate the effect size (d) and statistical power (P).

## 3. Results

The study was conducted in a total of 90 patients (90 eyes) with CM who met the inclusion criteria, and 90 healthy controls (90 eyes) matched by age and sex. The mean (SD) age was 42.3 ± 9.2 in the CM group and 41.6 ± 10.6 in the control group. The ages of the patients ranged from 18 to 65 years in both groups.

Both the CM patients and the control group consisted of 78 females (85.7%) and 12 males (14.3%). Regarding the laterality of their migraines, 54 patients (60%) reported a right-sided migraine pain preference, and 36 patients (40%) reported a left-sided predominance. In 16 of the study patients (17.8%), their CM occurred with a visual aura, of which 2 were males and 14 were females. There were no significant differences between the two groups in terms of age, sex, spherical equivalent, intraocular pressure, and visual acuity (*p* > 0.05). Table 1 summarises the socio-demographic and clinical data of the sample.

**Figure 3:**
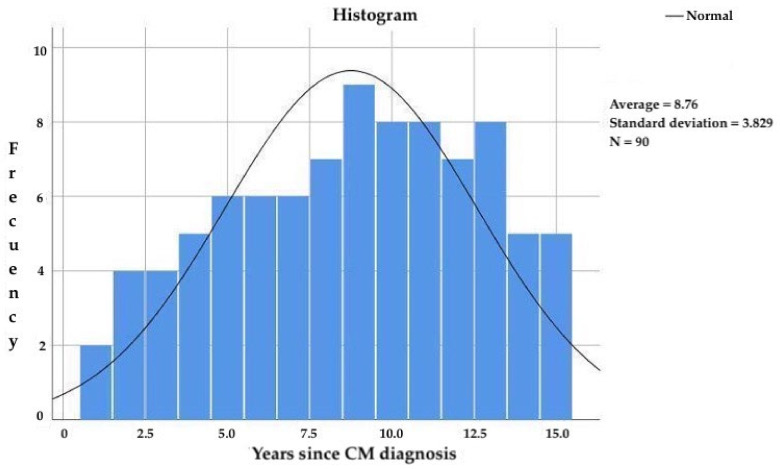
Years since CM diagnosis in the study participants.

The subjects included in the study had been diagnosed with CM for more than 3 years and less than 15 years, with a mean age of 8.8 ± 3.8 years (Figure 3) since CM diagnosis and 20.4 ± 10.3 years since migraine onset. The frequency of migraine attacks was 18.4 ± 4.7 attacks per month.

After the application of the MIDAS scale, the average score obtained was 38.5 ± 13.7, which means that according to this scale, the patients experienced a very serious impact on their quality of life. Of all patients with CM, 72 of them (80%) were undergoing some symptomatic treatment during the acute phase of pain: 38 patients were taking naproxen (42.2%); 22 patients were taking ibuprofen (24.4%); and 12 patients were taking paracetamol (13.3%). We excluded six subjects from the study whose medication, triptans, could affect the thickness of the retina as a result of its vasoconstrictor effect.

Forty-two patients were taking a preventive treatment (46.6%): 4 patients (4.4%) were taking beta-blockers (propranolol); 10 patients (11.1%) were taking calcium channel blockers (flunarizine); 14 patients (15.6%) were taking antiepileptics (10 patients took valproic acid, and 4 patients took topiramate); and 14 patients (15.6%) took antidepressants (amitriptyline). All patients in the study were taking 10 tablets of NSAIDs per month at most, for treatment in the acute phase of pain; therefore, no patient was considered to be abusing medication.

Statistically significant differences were found in the mean macular thickness, which was thinner in migraine patients (281.52 ± 12.41 µm) than in healthy controls ((286.09 ± 11.74 µm): t_178_ = 2.536; *p* = 0.012; IC 95% = (1.013; 8.122); d = 0.38; P = 0.81 (Table 2)).

Macular thickness was significantly lower (*p* ≤ 0.05) in the main quadrants (superior, inferior, peripheral inferior, temporal, and nasal) of migraine patients compared with the controls, except for the central quadrant (Table 3). When considering the Bonferroni correction, significant differences were only found in the superior quadrant (t_178_ = 3.213; *p* = 0.002; IC 95% = (2.542; 10.635); d = 0.48; *p* = 0.94), where there was an average effect size and high statistical power.

At the papilla level, no significant differences were found in the mean peripapillary RNFL thickness (t_178_ = 1.129; *p* = 0.261; IC 95% = (−1.170; 4.297); d = 0.17; *p* = 0.30, (Table 4)); however, we did find differences in the superior quadrant (t_178_ = 1.826; *p* = 0.036; IC 95% = (−0.327; 8.393); d = 0.27; P = 0.38). A thickening was found in the nasal sector, though it was not significant (Table 5).

As for the nerve fibre layer in the macular area (RNFL macular), in contrast to the peripapillary RNFL, we found significant differences in the mean value (t_178_ = 2.24; *p* = 0.026; IC 95% = (0.917; 1.451); d = 0.33; *p* = 0.72,) without taking the Bonferroni correction into consideration (Table 6). When we analysed the quadrants (Table 7), the greatest differences were found. Indeed, a medium effect size and a high statistical power were found in the following quadrants: peripheral superior, superior, peripheral inferior, nasal temporal, and nasal.

In the GCL, we found significant differences in the mean values for both quadrants: GCL++ (t_178_ = 2.540; *p* = 0.012; IC 95% = (0.645; 5.147); d = 0.38; *p* = 0.81) and GCL+ (t_178_ = 2.599; *p* = 0.010; IC 95% = (0.500; 3.659); d = 0.37; *p* = 0.80) (Table 6). Regarding the quadrants in both GCL++ (Table 8) and GCL+ (Table 9), we found significant differences in all except the peripheral temporal and inferior temporal quadrants for GCL++. Taking into account the Bonferroni correction, significant differences were found in the quadrants: GCL++: superior, t_178_ = 2.939; *p* = 0.004; IC 95% = (1.054; 5.367); d = 0.44; *p* = 0.90; GCL+: superior, t_178_ = 2.806; *p* = 0.006; IC 95% = (0.656; 3.766); d = 0.42; *p* = 0.88, and superior temporal, t_178_ = 2.707; *p* = 0.007; IC 95% = (0.632; 4.035); d = 0.41; *p* = 0.75.

### 3.1. Correlation between the Years with CM Diagnosis and Retinal Thickness

A statistical test of the Pearson correlation coefficient, which is conducted between different variables, was carried out between those layers where greater differences in thickness, macula, and GCL++ were found. This test was carried out in order to understand the relationship between the variables and the number of years since the patient had been diagnosed with CM.

**Figure 4:**
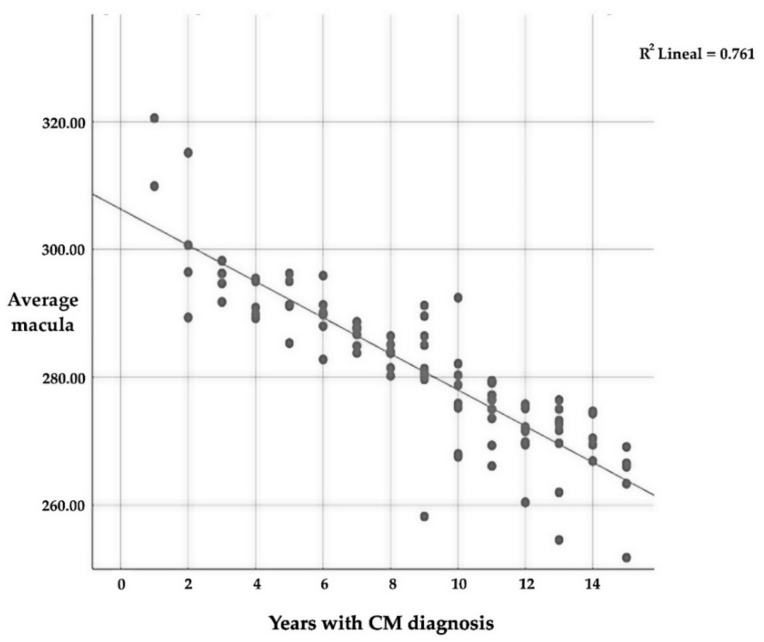
Correlation between average macular thickness and the number of years since CM diagnosis.

The mean macular thickness is intensely related (*p* ≤ 0.01) to the number of years since CM diagnosis, with a very strong negative correlation (r = −0.873); this implies that the longer one suffers from CM, the lower the average thickness of the macular (Table 10 and Figure 4). As for the relationship between the different quadrants, there is also a significant relationship (*p* ≤ 0.01) between all of them and the number of years that a patient suffers with CM.

Regarding the different quadrants, there is a significant correlation between them, except for the central one; in this case, the correlation is positive, which means that if thickness is reduced in one quadrant, in the other, the same thing will also happen. Table 10 only includes the average thickness and the number of years that the patient has suffered from CM in order to avoid representing a large amount of data.

**Figure 5:**
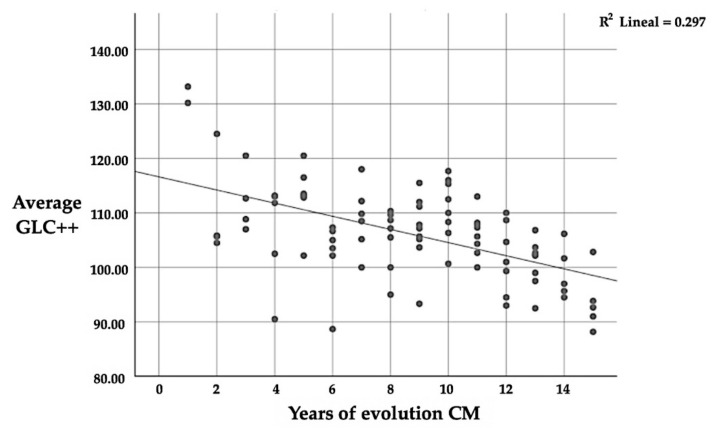
Correlation between GCL++ and the number of years since CM diagnosis.

Likewise, in the GCL++ layer, there is a significant correlation (*p* ≤ 0.01) between the number of years since CM diagnosis and thickness, with a mean negative correlation (r = −0.545); this implies that the longer one suffers from CM, the lower the average thickness of the GCL++ (Table 11 and Figure 5). This is the same as the correlation between the four quadrants and the average thickness; in this case, the correlation is positive again.

### 3.2. Relationship between Retinal Thickness and Pain Laterality regarding Chronic Migraines

No significant differences were found (*p* ≤ 0.05) after using Tukey’s post hoc correction for multiple comparisons. The differences between the mean thicknesses of the homolateral eye to migraine pain with the contralateral eye were contrasted, but no significant differences were found between the layers or quadrants of CM patients.

## 4. Discussion

Migraines predominantly affect the female sex [42], as was seen in the sample included in this study; the proportion of women in the sample was 85.7% compared with 14.3% men. The disease can begin at any age, although the first crisis usually occurs during adolescence, reaching its maximum incidence between 30–50 years of age, and it gradually becomes less intense and frequent in the following decades. In this study, the mean age of the patients was 42.3 ± 9.2 years.

In chronic migraine patients, differences in mean thickness were described in the macular, RNFL macular, and GCL (in both GCL++ and GCL+), which were significantly thinner than in healthy controls. Significant differences were also found in several quadrants of those same layers of the retina.

Statistically significant differences were found in the mean macular thickness (*p* ≤ 0.05), with a mean effect size (d = 0.38) that was thinner by 4.57 μm in patients with CM than in healthy controls. Macular thickness was lower in the main quadrants (superior, inferior, peripheral inferior, temporal, and nasal) of patients with CM, except for the central quadrant. Considering the Bonferroni correction (*p* ≤ 0.005), significant differences were only found in the superior quadrant, with a mean effect size (d = 0.48), and a high statistical power (P = 0.94). Other studies on changes in macular thickness in patients with migraines show contrasting results, as some studies find no differences between the patients and controls [13,43], whereas others report reductions in foveal thickness [44].

In our study, we found no significant differences in the mean thickness of the RNFL at the peripapillary level; however, the place where the greatest differences appear (4.03 μm), and the place with a small effect size (d = 0.27), is in the superior quadrant. Here, our findings are in accordance with the studies by Gipponi et al. [43] and Kirbas et al. [45], as they also only found differences in that quadrant; however, other studies report significant differences in the rest of the quadrants. Colak et al. [44] found a reduction in the thickness of the RFNL, in the superior quadrant, and in the inferior quadrant. Moreover, Dermican et al. [13] only found a difference in the nasal sector.

As for the layer of nerve fibres in the macular area (macular RNFL), unlike the peripapillary (peripapillary RNFL), significant differences (*p* ≤ 0.05) were found in the mean values, without taking the Bonferroni correction into consideration, with a small effect size (d = 0.33). When the different quadrants were analysed, the greatest differences were found in the peripheral superior, inferior, peripheral inferior, nasal temporal and nasal quadrants, with a medium effect size and high statistical power. These data could not be contrasted with other studies.

In the ganglion cell complex (GCC), in the average thickness of the layer called GCL++, we found a thickness difference of 2.91 μm. In the average thickness of the GCL+ layer there was a difference of 2.08 μm. Regarding the quadrants for both the GCL++ and GCL+ layers, significant differences were found in all sectors, except in the inferior temporal and superior temporal of the GCL++. All previously published studies confirm that the GCC is reduced in migraine patients [44,46,47], thus highlighting the data provided by Reggio et al. [48], which is the only study that included patients with CM.

These differences that were found between the different studies may be the consequence of the inequalities between the samples of each study, both in terms of the number of patients, the diversity of the population studied, the duration of the migraine, migraines with aura or without aura, and even by the collection of data, where different types of OCT were used. Most studies do agree that a significant reduction in the thickness of the RNFL exists, and that there is a correlation between thickness, the number of years that the patient has suffered with migraines, and the migraine’s severity.

Statistically significant differences have been found between migraine patients and control subjects in the retrobulbar circulation, which may imply greater ischaemia in the former [49]. These alterations in the vascular flow of the eyeball could also lead to a reduction of the GCL [36,46], and this may be the origin of visual field deficits observed in one third of these patients [50,51]. These deficits have been found in migraine patients both without aura and with visual aura. Moreover, these deficits tend to be more serious the day after a migraine crisis, and in many cases, they are still present a week later [52].

Although the symptomatology of visual auras in migraine is due to the cortical spreading depression that usually precedes the seizures [11], deficits in the intercritical periods have also been reported in different studies [53,54]. In all of them, altered perfusion in the optic nerve papilla is suggested as a plausible explanation.

The results obtained in these studies could be explained by the relationship between migraines and certain endothelial dysfunction, which would be considered as a systemic vasculopathy [55,56]. Interestingly, systemic vascular diseases also occur in patients with glaucoma and are considered vascular risk factors [57,58]. In fact, there are several studies that connect migraines with glaucoma [59,60], especially normotensive glaucoma, where a coexistence between migraines and glaucoma-like optic nerve damage has been found in subjects without any visual symptoms and with normal intraocular pressure. This has also been found in patients with visual field defects such as glaucomatous subjects [50,53]. This secondary hypoperfusion of the optic nerve could be the cause of the degeneration of the retinal ganglion cells and the consequent reduction of thickness in the different layers of the retina, both at the macular and peripapillary level. In addition, this relationship could be supported by the increasingly widespread hypothesis that the more chronic a migraine becomes, the more it behaves as a progressive degenerative disease with well-known anatomical changes at the brain level [10], and perhaps the ocular level as well.

In our study, after the application of the MIDAS scale, the average score obtained was 38.5 ± 13.7 points, which indicates very serious limitations of the quality of life of patients with CM, as well as important pharmacological treatment needs. In the work of Martínez et al. [36], in which they studied 70 patients with migraines against 53 healthy controls by comparing the thickness of the RNFL, the MIDAS scale was also applied, and the average score was 34.3 points; therefore, this study also found a strong correlation between the obtained score and the average thickness of the RNFL.

Another result obtained in our study was the close relationship (*p* ≤ 0.01) between the mean macular thickness, the GCC, and the number of years since CM diagnosis; indeed, a negative correlation was found, which implies that the longer one suffers from CM, the thinner the macular and GCC. As for the relationship between the different quadrants, there existed a significant relationship (*p* ≤ 0.01) between all of them and the number of years since CM diagnosis. In other studies, wherein they only studied episodic migraines and RNFL, they found no correlation between thickness reduction [43,47] and the number of years since migraine diagnosis. This could be because the correlation was not found in the macular area or in the GCC; according to the data obtained in our analysis, this is where the greater differences in thickness between patients and controls are found, and the type of migraine studied in that instance was an episodic migraine, not a CM.

Regarding the affectation of the thickness of the retina as a function of the laterality of the migraine, despite having found differences in thickness between the homolateral eye and migraine, and the contralateral eye and most of the analysed layers of the retina, this finding has no clinical relevance, since the differences found were not significant (*p* > 0.05). The data coincide with those provided by Gunes et al. [61], who, despite also finding differences in the thinning of the RNFL that was related to the lateralisation of the migraine, their results also did not find a statistical significance. If confirmed in subsequent studies, Gunes et al. point out that the relationship between the lateralisation of migraine pain and a greater loss of thickness in the same eye could be due to the decrease in blood flow from the retina in the homolateral eye as a result of the migraine.

In light of the results obtained in patients with CM, it would be interesting to continue this line of research, since OCT technology can be a very useful tool with which to evaluate the evolution of the disease. Indeed, this is due to the ease with which it measures the thickness of the different layers of the retina, and it can even check if preventive treatments can reduce or slow down the anomalies observed in the retinas of patients with CM.

## 5. Conclusions

In conclusion, the mean macular thickness, macular nerve fibre layer, and ganglion cell layer were significantly thinner in chronic migraine patients than in age and gender-matched healthy control subjects. We also found significant thickness differences in the main retinal quadrants when it was analysed by sector. No significant differences were found between the mean thicknesses of the peripapillary RNFL, although they did appear when analysed by quadrant; more specifically, significant differences appeared in the superior quadrant.

There is a strong negative correlation between the average thickness of the macula, the RNFL, and the number of years since the diagnosis of CM. On the contrary, no significant relationship was found between the laterality of the migraine and retinal involvement in any of the layers.

In light of the results, we believe that this line of research should be continued, as OCT technology, due to the ease with which it allows us to measure the thickness of the different layers of the retina, is a very useful tool that enables us to assess the evolution of the disease and check whether preventive treatments can reduce or slow down the abnormalities observed in the retina of patients with migraines.

## Figures and Tables

**Table 1:** Socio-demographic and clinical data of the CM patients and control subjects.

	CM Patients (*n* = 90)	Control Subjects (*n* = 90)
Age (years)	42.3 (9.2)	41.6 (10.6)
Males/Females	12 (14.3%)/78 (85.7%)	12 (14.3%)/78 (85.7%)
Intraocular pressure (mm Hg)	15.9 (2.1)	15.7 (1.2)
Spherical equivalent (dp)	0.19 (1.98)	0.15 (1.82)
Visual acuity (decimal)	0.96 (0.18)	1.06 (0.05)
Evolution years EM	20.4 (10.3)	
Evolution years CM	8.8 (3.8)	
Laterality CM	54 patients (60%) right 36 patients (40%) left	
Visual aura	14 women (15.6%) 2 men (2.2%)	
Attacks per month	18.4 (4.7)	
MIDAS scale	38.5 (13.7) grade IV	

Mean values (standard deviation). EM: episodic migraine. CM: chronic migraine. MIDAS: migraine disability assessment scale.

**Table 2:** Average macular thickness (μm).

Control Subjects *n* = 90	SD Control (±)	Migraine Patients *n* = 90	SD Migraine (±)	*p* Value
286.09	11.74	281.52	12.41	**0.012**

Mean (SD) value of the macular thickness (μm) in each of the 90° quadrants around the macula of the control subjects and chronic migraine patients. Significance of difference in the *t*-test: *p*-value. Statistically significant data is in bold (*p* ≤ 0.05).

**Table 3:** Macular thickness (μm) in each of the 90° quadrants around the macula.

Quadrants (µm)	Control Subjects *n* = 90	SD Control (±)	Migraine Patients *n* = 90	SD Migraine (±)	*p* Value
Peripheral superior	275.49	14.61	272.02	13.70	0.102
Superior	315.33	12.50	308.74	14.90	0.002 *
Central	239.52	18.45	234.12	21.13	0.070
Inferior	313.14	12.42	307.42	15.15	**0.006**
Peripheral inferior	267.80	16.47	261.87	13.55	**0.009**
Peripheral temporal	257.81	14.28	254.93	12.83	0.157
Temporal	301.07	11.95	296.26	15.32	**0.020**
Nasal temporal	289.13	15.65	285.72	14.86	0.131
Nasal	315.84	12.89	310.08	15.68	**0.008**

Mean (SD) value of the macular thickness (μm) in each of the 90° quadrants around the macula of control subjects and chronic migraine patients. Significance of difference in the *t*-test: *p*-value. Statistically significant data is in bold (*p* ≤ 0.05). * Considering the Bonferroni correction (*p* ≤ 0.005).

**Table 4:** Average peripapillary RNFL thickness (μm).

Control Subjects *n* = 90	SD Control (±)	Migraine Patients *n* = 90	SD Migraine (±)	*p* Value
106.53	9.36	104.96	9.23	0.261

Mean (SD) value of the average peripapillary RNFL thickness (μm) for each of the 90° quadrants of the control subjects and chronic migraine patients. Significance of difference in the *t*-test: *p*-value.

**Table 5:** Peripapillary RNFL thickness (μm) in each of the 90° quadrants around the optic disk.

Quadrants (µm)	Control Subjects *n* = 90	SD Control (±)	Migraine Patients *n* = 90	SD Migraine (±)	*p* Value
Superior	129.90	15.43	125.87	14.19	**0.036**
Inferior	136.81	15.23	133.31	14.65	0.118
Temporal	74.24	11.81	72.57	11.20	0.329
Nasal	74.80	14.61	75.69	12.57	0.662

Mean (SD) value of the average peripapillary RNFL thickness (μm) for each of the 90° quadrants of the control subjects and chronic migraine patients. Significance of difference in the *t*-test: *p*-value. Statistically significant data is in bold (*p* ≤ 0.05).

**Table 6:** RNFL macular, GCL++, and GCL+ thickness (μm).

Quadrants (µm)	Control Subjects *n* = 90	SD Control (±)	Migraine Patients *n* = 90	SD Migraine (±)	*p* Value
RNFL macular	28.90	2.01	28.13	2.58	**0.026**
GCL++ ^1^	108.76	6.55	105.85	8.61	**0.012**
GCL+ ^2^	73.76	5.20	71.68	5.53	**0.010**

^1^ Ganglion cell complex (GCC): RNFL + ganglion cell layer (GCL) + inner plexiform layer (IPL). ^2^ Ganglion cell layer + inner plexiform layer (GCIP): GCL + IPL. Significance of difference in the *t*-test: *p*-value. Statistically significant data is in bold (*p* ≤ 0.05).

**Table 7:** RNFL macular thickness (μm) in each of the 90° quadrants around the macula.

Quadrants (µm)	Control Subjects *n* = 90	SD Control (±)	Migraine Patients *n* = 90	SD Migraine (±)	*p* Value
Peripheral superior	40.08	3.78	38.79	3.95	**0.026**
Superior	27.32	2.25	26.80	2.25	0.121
Central	3.94	2.19	3.60	2.67	0.345
Inferior	28.41	4.07	27.27	2.41	**0.023**
Peripheral inferior	41.77	3.67	40.38	5.54	**0.050**
Peripheral temporal	22.89	2.43	22.91	2.50	0.952
Temporal	20.68	1.87	20.22	1.92	0.109
Nasal temporal	51.52	5.05	48.89	2.63	**0.014**
Nasal	23.82	1.91	23.11	2.31	**0.026**

Mean (SD) value of the RNFL macular thickness (μm) in each of the 90° quadrants around the macula of control subjects and chronic migraine patients. Significance of difference in the *t*-test: *p*-value. Statistically significant data is in bold (*p* ≤ 0.05).

**Table 8:** GCL++ thickness (μm) in each of the 90° quadrants around the macula.

Quadrants (µm)	Control Subjects *n* = 90	SD Control (±)	Migraine Patients *n* = 90	SD Migraine (±)	*p* Value
Superior	109.83	6.97	106.61	7.67	0.004 *
Superior temporal	96.46	6.00	94.11	7.26	0.085
Superior nasal	118.17	13.55	113.84	16.09	**0.050**
Inferior	107.81	6.96	105.00	9.01	**0.021**
Inferior temporal	99.56	8.45	97.46	11.70	0.169
Inferior nasal	121.93	7.19	118.46	11.16	**0.014**

Mean (SD) value of the average peripapillary GCL++ thickness (μm) for each of the 90° quadrants of the control and patient groups. Significance of difference in the *t*-test: *p*-value. Statistically significant data is in bold (*p* ≤ 0.05). * Considering the Bonferroni correction (*p* ≤ 0.007).

**Table 9:** GCL+ thickness (μm) in each of the 90° quadrants around the macula.

Quadrants (µm)	Control Subjects *n* = 90	SD Control (±)	Migraine Patients *n* = 90	SD Migraine (±)	*p* Value
Superior	73.26	5.17	71.04	5.40	0.006 *
Superior temporal	72.50	5.42	70.17	6.13	0.007 *
Superior nasal	76.38	5.94	74.52	5.84	**0.036**
Inferior	70.42	5.25	68.37	5.44	**0.011**
Inferior temporal	74.63	5.54	72.32	6.69	**0.012**
Inferior nasal	75.94	5.75	73.92	5.63	**0.018**

Mean (SD) value of the average peripapillary GCL+ thickness (μm) for each of the 90° quadrants of the control and patient groups. Significance of difference in the *t*-test: *p*-value. Statistically significant data is in bold (*p* ≤ 0.05). * Considering the Bonferroni correction (*p* ≤ 0.007).

**Table 10:** Correlation between the number of years since the patient received a CM diagnosis and the mean macular thickness.

		Average Macula	Years
Average macula	Pearson correlation	1	−0.873 *
	Sig. (bilateral)		<0.01
	n	90	90
Years	Pearson correlation	−0.873 *	1
	Sig. (bilateral)	<0.01	
	n	90	90

* The correlation is significant at level 0.01 (bilateral).

**Table 11:** Correlation between the number of years since CM diagnosis and the average thickness of the GCL++.

		Average GCL++	Years
Average GCL++	Pearson correlation	1	−0.545 *
	Sig. (bilateral)		<0.01
	n	90	90
Years	Pearson correlation	−0.545 *	1
	Sig. (bilateral)	<0.01	
	n	90	90

* The correlation is significant at level 0.01 (bilateral).

## Data Availability

Not applicable.
